# Outcomes Associated With the Timing of Antivenom Administration in *Bothrops* and *Crotalus* Snakebite Envenomations

**DOI:** 10.1590/0037-8682-0588-2025

**Published:** 2026-07-03

**Authors:** Rafaele Maria Tirolla, Edmarlon Girotto, Alberto Duran Gonzalez, Camilo Molino Guidoni

**Affiliations:** 1 Universidade Estadual de Londrina, Programa de Pós-Graduação em Ciências Farmacêuticas, Londrina, PR, Brasil.; 2 Universidade Estadual de Londrina, Programa de Pós-Graduação em Saúde Coletiva, Londrina, PR, Brasil.

**Keywords:** Poison Control Centers, Snake Bites, Treatment Outcome, Venomous Snakes

## Abstract

**Background:**

Snakebite envenomation is a significant public health problem. We aimed to analyze the clinical outcomes associated with the timing of antivenom administration in *Bothrops* and *Crotalus* snakebite envenomations.

**Methods:**

This descriptive, retrospective, census-based cohort study included patients who attended the Toxicological Information and Assistance Center-Londrina from 2017 to 2024. Sociodemographic, clinical, exposure-related, and antivenom therapy data were obtained from the national DATATOX system and clinical records. Descriptive statistics and Poisson regression (for relative risks [RRs] and 95% confidence intervals [CIs]) were used; significance was set at p<0.05.

**Results:**

Data from 1,162 cases were analyzed, with 72.8% caused by *Bothrops* and 27.2% by *Crotalus*. Most patients were male (78.6%), rural workers (59.0%), and residents of rural areas (88.4%). Antivenom therapy was administered with 98.2% adequacy, according to institutional protocols. The median time from bite to completion of antivenom infusion was 450 minutes, with 75% of patients treated within 12 hours; 2.7% of patients developed renal complications and 1.1% died. Both outcomes were more frequent among patients with longer intervals between the bite and antivenom administration (>75th percentile). In the crude analysis, delays were associated with renal complications (RR: 3.21; 95% CI: 1.61-6.41; p=0.001) and death (RR: 4.81; 95% CI: 1.59-14.59; p=0.006). After adjustment, the association remained significant for renal complications (RR: 2.88; 95% CI: 1.37-6.06; p=0.005), but not for death.

**Conclusions:**

Delayed antivenom administration is associated with an increased risk of renal complications, reinforcing the timing of therapy as a critical and modifiable factor influencing snakebite outcomes.

## INTRODUCTION

Snakebite envenomation is a significant public health concern due to its high incidence and potential severity. Its disproportionate impact on socioeconomically vulnerable populations has led to the World Health Organization recognizing it as one of the neglected tropical diseases^1^ ^-^ ^3^. In Brazil, snakebite envenomation is currently included in the National Compulsory Notification List^4^. In 2024, 334,561 envenomation incidents caused by venomous animals were reported in Brazil through the Notifiable Diseases Information System; of these 31,464 (9.4%) were attributed to snakebites. In the southern region of Brazil, 2,549 cases of snakebite envenomation were reported, including 922 cases in the state of Paraná^5^. 

In Brazil, medically important snakebite envenomations are classified into four types: bothropic, crotalic, lachestic, and elapidic. Among these, bothropic (*Bothrops jararaca, B. alternatus, B. jararacussu, B. neuwiedi*) and crotalic (*Crotalus durissus*) envenomations are particularly noteworthy because their incidence in the southern region of the country is high^2^ ^,^ ^6^.

Species of the genus *Bothrops* are widely distributed across Brazil and well adapted to anthropized environments, which contributes to the high incidence of *Bothrops* envenomations of humans^2^. Their venom is predominantly composed of metalloproteinases, phospholipase A2, and serine proteases, which are responsible for the proteolytic, hemorrhagic, and inflammatory effects^7^ ^,^ ^8^. Clinically, bothropic envenomation is characterized by prominent local manifestations such as pain, edema, ecchymosis, and tissue necrosis, in addition to systemic complications that include coagulopathy, hemorrhagic events, and kidney injury^6^ ^-^ ^8^.

Conversely, snakes of the genus *Crotalus*, commonly known as rattlesnakes and characterized by the presence of a rattle at the tail, are generally less aggressive but associated with more severe systemic symptoms of envenomation^2^. Their venom contains components such as crotoxin, which exerts neurotoxic and myotoxic effects by impairing neuromuscular transmission^9^. Crotalic envenomation therefore typically presents with mild local findings but marked systemic involvement. This involvement includes neurotoxic manifestations such as ptosis, blurred vision, and ophthalmoplegia as well as myotoxicity, which leads to generalized muscle pain, dark urine, and a high risk of acute kidney injury^10^ ^,^ ^11^.

In this context, the Toxicological Information and Assistance Centers (CIAToxs) and Brazilian Toxic Exposure Database (DATATOX) constitute authoritative sources that provide standardized clinical and epidemiological data for supporting retrospective analyses. This study leveraged integration of the Toxicological Information and Assistance Center of Londrina (CIATox-Londrina) records with the DATATOX to assemble a retrospective cohort for evaluating the association between latency to antivenom administration and key clinical endpoints, including acute kidney injury and mortality. 

Studies examining these factors are essential to support local and regional interventions, such as optimizing the logistics of antivenom distribution and organizing care pathways at reference centers to manage these events. Such strategies may contribute to reducing the morbidity and mortality associated with snakebite envenomation and improving the quality of patient care. Accordingly, in the present study, we analyzed the data of patients envenomated by snakes of the genera *Bothrops* and *Crotalus* who attended a CIATox. By conducting this analysis, we aimed to describe the sociodemographic and clinical profiles of these patients and investigate the outcomes associated with the timing of antivenom administration.

## METHODS

This retrospective, descriptive, census-based cohort study included patients who suffered snakebite envenomation and were attended to at the CIATox-Londrina between January 2017 and December 2024. As a census-based study, all cases recorded during this period were included, precluding the need to perform a sample size calculation. CIATox-Londrina, located at the University Hospital of the State University of Londrina, serves as a toxicology reference center for the northern macroregion of Paraná state. CIATox centers are public service centers specializing in clinical toxicology; they provide technical and clinical support for cases of poisoning and envenomation by venomous animals, operate continuously, and offer both in-person and remote assistance to healthcare professionals and the general population. These centers are staffed by multidisciplinary teams, enabling an integrated approach to patient management. This study was conducted in accordance with the Strengthening the Reporting of Observational Studies in Epidemiology guidelines^12^.

The study population comprised all patients who suffered snakebite envenomation by snakes of the genera *Bothrops* and *Crotalus* and were attended at CIATox-Londrina during the study period. Data were obtained from standardized clinical records in the DATATOX, a database adopted by the center in 2017. Records of patients who were simultaneously managed at another CIATox, which subsequently continued the patient’s follow-up, were excluded.

Data were collected using DATATOX, a national database of poisoning and envenomation cases maintained by the Brazilian Association of Toxicological Information and Assistance Centers. Implemented in 2014, the system enables standardized recording of clinical and epidemiological information and includes an analytical module (DATATOX-BI) that is used to facilitate the generation of statistical reports and epidemiological analyses, ensuring data reliability and traceability. In addition to being extracted via DATATOX-BI, the clinical records of patients were individually reviewed to supplement relevant temporal information. No additional strategies to control for bias were applied beyond standardized data collection and exclusion of duplicate records; we therefore acknowledge that potential bias due to incomplete records and clinical variability among patients’ originating centers, particularly regarding initial management and antivenom availability, may exist.

The variables analyzed included sociodemographic characteristics (sex, age, age group, race/ethnicity, education level, and occupation), exposure-related factors (area of occurrence, anatomical site of the bite, use of a tourniquet, snake genus, animal identification, and time elapsed from envenomation to initial care), and clinical characteristics (presence of symptoms, need for and duration of hospitalization, hospital ward, final severity classification, and adverse outcomes of interest for subsequent analysis of associations, including death and renal complications). The variable *renal complications* encompassed patients who developed acute kidney injury or required hemodialysis. Antivenom-related variables were also assessed, including antivenom use and appropriateness, need for additional doses, occurrence of adverse reactions, time from envenomation to completion of infusion, and time from indication to completion of therapy. Antivenom therapy was considered inadequate if the type of antivenom administered differed from that recommended or if the number of vials used was less than that recommended in the CIATox-Londrina guidelines. Time elapsed from envenomation to completion of antivenom administration was initially treated as a continuous variable and subsequently categorized into quartiles. For association analysis, two groups were established: one comprising patients with an antivenom administration time of ≤75th percentile and the other comprising those with an antivenom administration time of >75th percentile to evaluate the relationship between this time and adverse clinical outcomes.

Data were organized and tabulated in electronic spreadsheets and analyzed using the Statistical Package for the Social Sciences (SPSS), version 26.0. Categorical variables are expressed as absolute and relative frequencies, whereas continuous variables are summarized using measures of central tendency and dispersion. Associations between covariates (sex, age group, snake genus, antivenom appropriateness, and snake identification) and clinical outcomes (death and renal complications) were evaluated using Pearson’s chi-square or Fisher’s exact test, as appropriate. The association between time to antivenom completion and adverse clinical outcomes was analyzed using Poisson regression with robust variance estimation (sandwich estimator) to directly estimate relative risks (RRs), with both crude and adjusted models constructed; the latter included variables of prior clinical and epidemiological relevance. Results were considered statistically significant at a p-value of <0.05, with a 95% confidence interval (CI).

A post hoc sample size power analysis was performed, considering a significance level of 5% and 95% confidence interval, to assess the ability of the study to detect associations between the time from bite to completion of antivenom infusion and the outcomes analyzed.

This study was approved by the Research Ethics Committee of the State University of Londrina (approval number CAAE 45986415.1.0000.5231, opinion number 6.140.867).

## RESULTS

Between January 2017 and December 2024, 1,368 cases of snakebite envenomation by snakes of the genera *Bothrops* and *Crotalus* were attended to at CIATox-Londrina. After excluding 44 duplicate records or cases referred to other centers, 1,324 cases remained eligible for analysis. An additional 162 records were excluded due to missing information that prevented the time required to complete antivenom serotherapy from being determined. Thus, the study population comprised 1,162 cases of envenomation caused by snakes of the *Bothrops* and *Crotalus* genera.

Most patients were male (78.6%), and their ages ranged from 20 to 59 years (64.5%). Patients were primarily white (70.2%), had a primary school education (59.7%), and engaged in rural occupations (59.0%) (Table 1). The median age of the cohort was 47 (interquartile range, 29; minimum, 1 year; maximum, 94 years).

**TABLE 1:** Sociodemographic characteristics of patients with *Bothrops* or *Crotalus* envenomation who attended the Toxicological Information and Assistance Center of Londrina, from January 2017 to December 2024.

Sociodemographic variable	Total	Age range		
		≤19 years old	20-59 years old	≥60 years old
	n (%)	n (%)	n (%)	n (%)
**Sex (N=1,162)**				
Male	913 (78.6)	102 (73.4)	595 (79.4)	216 (78.8)
Female	249 (21.4)	37 (26.6)	154 (20.6)	58 (21.4)
**Race/Ethnicity (N=1,154)**				
White	810 (70.2)	79 (57.7)	516 (69.4)	215 (78.8)
Mixed	268 (23.2)	38 (27.7)	188 (25.3)	42 (15.4)
Black	39 (3.4)	5 (3.6)	24 (3.2)	10 (3.7)
Indigenous	29 (2.5)	14 (10.2)	13 (1.7)	2 (0.7)
Asian	8 (0.7)	1 (0.7)	3 (0.4)	4 (1.5)
**Education level (N=1,101)**				
Illiterate	38 (3.5)	-	15 (2.1)	23 (8.9)
Incomplete primary education	389 (35.3)	66 (55.5)	207 (28.6)	116 (45.0)
Complete primary education	230 (20.9)	7 (5.9)	160 (22.1)	63 (24.4)
Incomplete secondary education	95 (8.6)	34 (28.6)	47 (6.5)	14 (5.4)
Complete secondary education	315 (28.6)	9 (7.6)	272 (37.6)	34 (13.2)
Incomplete higher education	8 (0.7)	3 (2.5)	4 (0.6)	1 (0.4)
Complete higher education	26 (2.4)	-	19 (2.6)	7 (2.7)
**Occupation (N=1,116)**				
Rural worker	658 (59.0)	17 (14.4)	491 (67.4)	150 (55.8)
Retired	99 (8.9)	-	13 (1.8)	86 (32.0)
Student	97 (8,7)	94 (79.7)	3 (0.4)	-
Homemaker	69 (6.2)	-	53 (7.3)	16 (5.9)
Construction worker	28 (2.5)	1 (0.8)	24 (3.3)	3 (2.5)
Unemployed	22 (2.0)	3 (2.5)	18 (2.5)	1 (0.4)
General services	19 (1.7)	-	16 (2.2)	3 (1.1)
Domestic worker	9 (0.8)	-	9 (1.2)	-
Other	115 (10.3)	3 (2.5)	102 (14.0)	10 (3.7)

Most envenomation incidents occurred in rural areas (88.4%), with the feet (39.8%), legs (28.0%), and hands (26.1%) being the most frequently affected anatomical sites. Bothropic envenomations accounted for 72.8% of cases, and the snake was identified in 48.4% of incidents, either by photograph or by presenting the animal at the center (Table 2). Tourniquet use was observed in only 0.8% of cases. The median time from envenomation to initial care was 60 minutes (range: 10-4,320 minutes [3 days]).

**TABLE 2:** Characteristics of *Bothrops* and *Crotalus* snakebite incidents involving patients treated at the Toxicological Information and Assistance Center of Londrina, from January 2017 to December 2024.

Variable	Total	Time from bite to completion of antivenom infusion	
	n (%)	≤75th percentile	>75th percentile
		n (%)	n (%)
**Exposure area (N=1,160)**			
Rural	1,025 (88.4)	775 (89.1)	250 (86.2)
Urban	120 (10,3)	83 (9.5)	37 (12.8)
Peri-urban	15 (1.3)	12 (1.4)	3 (1.3)
**Anatomical site of bite (N=1,320)**			
Foot	462 (39.8)	345 (39.6)	117 (40.3)
Leg	325 (28.0)	235 (27.0)	90 (31.0)
Hand	303 (26.1)	240 (27.6)	63 (21.7)
Arm	45 (3.9)	30 (3.4)	15 (5.2)
Other sites	26 (2.2)	21 (2.4)	5 (1.7)
**Snake genus (N=1,162)**			
Bothrops	846 (72.8)	668 (76.6)	178 (61.4)
Crotalus	316 (27.2)	204 (23.4)	112 (38.6)
**Snake identification (N=1,162)**			
Identified	562 (48.4)	461 (52,9)	101 (34,8)
Not identified	600 (51.6)	411 (47.1)	189 (65.2)

All patients evaluated presented with clinical manifestations, often more than one per individual. Among bothropic envenomations, the most frequent manifestations were pain (94.8%) and edema (94.6%), followed by bite marks (77.3%), local bleeding (46.2%), and hypertension (29.6%). Among crotalic envenomations, pain (76.9%), blurred vision (68.7%), bite marks (60.4%), edema (49.7%), and palpebral ptosis (52.2%) predominated (Table 3).

**TABLE 3:** Main clinical manifestations of *Bothrops* and *Crotalus* snakebites in patients treated at the Toxicological Information and Assistance Center of Londrina, from January 2017 to December 2024.

Clinical manifestations	n	%
***Bothrops* (N=846)**		
Pain	802	94.8
Edema	800	94.6
Fang/bite marks	654	77.3
Local bleeding	391	46.2
Hypertension	250	29.6
Ecchymosis	225	26.6
Hyperemia	150	17.7
Hematoma	141	16.7
Tachycardia	103	12.2
***Crotalus* (N=316)**		
Pain	243	76.9
Blurred vision	217	68.7
Fang/bite marks	191	60.4
Edema	157	49.7
Ptosis	165	52.2
Hypertension	118	37.3
Paresthesia	93	29.4
Generalized myalgia	87	27.5
Dizziness/vertigo	63	19.9

Of all the patients attended to, 74.9% (n=870) required admission to hospital, with a median length of stay of 2 days (interquartile range: 1-3 days; range: 0-41 days). Most admissions were to the emergency department (58.9%), followed by the ward (19.0%) and urgent care unit (11.6%); 10.0% of patients required intensive care. The final severity classifications showed that cases classified as mild (34.5%) and moderate (43.9%) predominated, followed by classifications of severe (19.6%), fatal (1.1%), and with no recorded outcome (0.9%).

Most patients (approximately 80%) received initial care from health services within 3 hours. According to CIATox-Londrina guidelines, 98.2% of patients were appropriately administered antivenom. Among treated individuals, 12.0% required an additional dose of antivenom, and 1.3% had an adverse reaction to the antivenom. The median time from indication to completion of antivenom infusion was 180 minutes, with 75% of patients completing antivenom therapy within 4 hours. The total time from envenomation to completion of antivenom therapy was 450 minutes. In most cases (75%), infusion was completed within 12 hours of the incident. In 290 patients, the antivenom administration time was above the 75th percentile (>P75).

Regarding adverse clinical outcomes of interest, 13 patients (1.1%) died, with the deaths equally distributed in absolute numbers between *Bothrops* (n=6; 0.7%) and *Crotalus* (n=7; 2.2%) envenomations. Thirty-one cases (2.7%) of renal complications were identified, of which 16 resulted from *Bothrops* bites (1.9%) and 15 from *Crotalus* bites (4.7%). Twelve of the thirty-one patients in these cases presented with renal impairment and underwent hemodialysis: two underwent hemodialysis only, and seventeen presented with renal kidney only.

In the analysis using adjusted variables, a significant association was observed between death and age group (p=0.035), snake genus (p=0.037), and adequacy of antivenom therapy (p=0.004), indicating a higher risk of mortality among adults aged 60 or older, those bitten by snakes of the genus *Crotalus*, and patients who received inadequate treatment. Regarding the occurrence of renal complications, significant associations were found with age group (p<0.001) and the genus of the snake involved (p=0.007), showing a higher proportion of cases among older individuals and those bitten by snakes of the genus *Crotalus* (Table 4).

**TABLE 4:** Association between the adjusted variables and clinical outcomes of death and renal complications of patients with *Bothrops* or *Crotalus* snakebite envenomation treated at the Toxicological Information and Assistance Center of Londrina, from January 2017 to December 2024.

Adjusted variable	Category	Death, n (%)		p-Value*	Renal complications, n (%)		p-Value*
		Yes	No		Yes	No	
**Sex**	Male	10 (1.1)	903 (98.9)	0.551	22 (2.4)	891 (97.6)	0.296
	Female	3 (1.2)	246 (98.8)		9 (3.6)	240 (96.4)	
**Age group**	≤19 years	1 (0.7)	138 (99.3)	0.035	1 (0.7)	138 (99.3)	<0.001
	20-59 years	5 (0.7)	744 (99.3)		10 (1.3)	739 (98.7)	
	≥60 years	7 (2.6)	267 (97.4)		20 (7.3)	254 (92.7)	
**Snake genus**	*Bothrops*	6 (0.7)	840 (99.3)	0.037	16 (1.9)	830 (98.1)	0.007
	*Crotalus*	7 (2.2)	309 (97.8)		15 (4.7)	301 (95.3)	
**Adequacy of antivenom therapy**	Adequate	19 (1.7)	1.130 (98.3)	0.022	30 (2.6)	1.111 (98.2)	0.436
	Inadequate	2 (15.4)	11 (84.6)		1 (3.2)	20 (96.8)	
**Snake identification**	Identified	5 (0.9)	557 (99.1)	0.472	15 (2.7)	547 (97.3)	0.998
	Not identified	8 (1.3)	592 (98.7)		16 (2.7)	584 (97.3)	

*Pearson’s chi-square or Fisher’s exact test.

A relationship was observed between longer time intervals from the bite to completion of antivenom infusion and worse clinical outcomes. The mortality rate among patients who received antivenom within a shorter time interval (P≤75%) was 0.6% (n=5), compared with the rate of 2.8% (n=8) among those who received antivenom after a longer interval (P>75%). A similar trend was observed for renal complications (1.7%, n=15 vs. 5.5%, n=16) (Table 5).

**TABLE 5:** Association between time from bite to completion of antivenom infusion and clinical outcomes in patients with *Bothrops* or *Crotalus* snakebite envenomation treated at the Toxicological Information and Assistance Center of Londrina, from January 2017 to December 2024.

Time from bite to completion of antivenom infusion	Death			Renal complication		
	n (%)	Crude analysis RR	Adjusted analysis	n (%)	Crude analysis, RR	Adjusted analysis,
		(95% CI);	RR (95% CI);		(95% CI);	RR (95% CI);
		p-value*	p-value*		p-Value*	p-Value*
**≤75th percentile (N=872)**	5 (0.6)	1.00	1.00	15 (1.7)	1.00	1.00
**>75th percentile (N=290)**	8 (2.8)	4.81 (1.59-14.59); 0.006	2.37 (0.54-10.44); 0.253	16 (5.5)	3.21 (1.61-6.41); 0.001	2.88 (1.37-6.06); 0.005

*Poisson regression with robust variance estimation (sandwich estimator). Adjusted analysis: adjusted for sex, age group, snake genus, adequacy of antivenom therapy, and snake identification. **RR:** Relative Risk; **CI:** Confidence Interval.

In the Poisson regression analysis, a longer time to antivenom infusion was associated with worse clinical outcomes. In the crude analysis, a significant association was observed between times greater than the 75th percentile and the occurrence of renal complications (RR: 3.21; 95% CI: 1.61-6.41; p=0.001) and death (RR: 4.81; 95% CI: 1.59-14.59; p=0.006). After adjusting for sex, age group, snake genus, adequacy of antivenom therapy, and snake identification, the association remained significant for the occurrence of renal complications (RR: 2.88; 95% CI: 1.37-6.06; p=0.005), although no significant association was observed between longer time to antivenom infusion and death (Table 5).

The results of the post hoc analysis indicated that the statistical power for detecting the association between the time from bite to completion of antivenom infusion and renal complications was adequate (90.67%), and the power for detecting the outcome of death was moderate to adequate (77.39%).

## DISCUSSION

The sociodemographic profile of patients included in this study is consistent with national epidemiological data on snakebite envenomation, namely, that affected individuals are predominantly male, of productive age, rural workers, have low educational attainment, and were exposed to the snake in rural areas^13^ ^-^ ^16^. The predominance of incidents that occurred in rural areas reflects the vulnerability and greater exposure that this population has to risk zones and agricultural activities, which increase the likelihood of contact with venomous snakes^14^ ^,^ ^17^. These findings highlight the public health relevance of snakebites and their close relationship with agricultural work. The predominance of *Bothrops* envenomations is consistent with the distribution of venomous snakes across Brazil and with data from other regions of Latin America^1^ ^,^ ^14^ ^,^ ^18^ ^-^ ^19^. Recent Brazilian studies have reinforced this epidemiological profile, demonstrating similar patterns across different regions of the country, including a predominance of *Bothrops* envenomations and higher incidence among rural male populations. Furthermore, a seasonal pattern in snakebite incidence, typically associated with warmer and rainier periods, is consistently reported in the literature^20^ ^-^ ^22^.

The anatomical sites most frequently affected by snakebites were the lower limbs, particularly the feet and legs; this is a consistent finding in the literature^15^ ^,^ ^23^. This pattern is related to the animal’s behavior and to the fact that most exposures occur during activities involving ground-level movement or agricultural work^18^ ^,^ ^23^ ^,^ ^24^.

In more than half of the cases, the snake species was accurately identified. This is a relevant finding, as this practice reduces diagnostic errors and contributes to appropriate clinical management. Correct species identification directly impacts the selection of therapeutic approaches and overall management of patients with envenomation. A residual proportion of diagnoses based solely on clinical evaluation confirms the importance of continuously training healthcare professionals to improve diagnostic accuracy and case management^25^ ^-^ ^27^, particularly considering that the clinical manifestations of different types of envenomation may overlap.

The high frequency of patients in this study who received initial care from health services within 3 hours after the snakebite indicates that the regional healthcare network has a certain degree of effectiveness, although delays beyond this period remain clinically relevant. Timely medical care is directly related to prognosis, with large-scale national studies reporting that delays in antivenom administration are associated with increased severity of envenomation. A national study analyzed 144,251 snakebite cases in Brazil between 2007 and 2015, demonstrating that delaying the administration of antivenom therapy by ≥6 hours after the bite was strongly associated with greater severity for both *Bothrops* and *Crotalus* envenomation^28^. Similarly, a study conducted in the Amazon region that included 9,191 snakebite cases reported that medical treatment initiated >6 hours after the bite was independently associated with a higher risk of severe outcomes and mortality^14^.

The main signs and symptoms observed in both types of envenomation are consistent with those reported in the literature. *Bothrops* envenomation was most frequently associated with intense pain, edema, ecchymosis, blistering, and local necrosis, resulting from the proteolytic and hemorrhagic actions of the venom^29^ ^,^ ^30^. *Crotalus* envenomation, predominantly neurotoxic and myotoxic with mild local effects, was generally related to manifestations such as ptosis, muscle weakness, and acute renal failure^31^ ^,^ ^32^.

Consistent with the findings of previous studies, the most frequent final classifications of case severity were mild and moderate^15^ ^,^ ^28^ ^,^ ^23^. Similarly, a study in the state of Rio Grande do Norte in which 3,019 snakebite cases were analyzed reported a case-fatality rate of <1%^23^. The availability of specific antivenoms and standardized clinical management protocols contribute to these favorable outcomes^2^ ^,^ ^33^. Therefore, the low proportion of severe and fatal cases observed in the present study reinforces the effectiveness of timely and adequate specific treatment, as well as the importance of its decentralization and accessibility.

Hospitalized patients had short hospital stays, with most being admitted to the general ward and having a low need for intensive care support. This finding is consistent with the predominance of mild and moderate clinical presentations observed in this study. Similarly, other investigations have reported a low frequency of severe complications and, consequently, shorter hospitalization times^28^ ^,^ ^34^. This pattern may reflect the effectiveness of CIATox-guided clinical management and the regional availability of antivenom therapy.

The high rate of antivenom use and appropriate administration observed in this study is consistent with findings from other national investigations^23^ ^,^ ^28^ ^,^ ^33^ ^,^ ^35^ ^,^ ^36^, which also reported that omission of antivenom therapy is uncommon and typically limited to asymptomatic individuals. Regarding treatment safety, severe adverse reactions to antivenom were rare and generally manageable with adequate hospital support^28^ ^,^ ^33^ ^,^ ^35^ ^,^ ^37^. CIATox-Londrina previously adopted an institutional protocol that included medications to prevent adverse reactions. This practice was inconsistent throughout the study period and discontinued in January 2024. Therefore, the low rate of adverse reactions cannot be solely attributed to these measures and may also reflect improvements and standardization of infusion protocols.

In the present study, most patients completed antivenom infusion within 4 hours after indication. However, when considering the time interval between the bite and completion of antivenom administration, antivenom infusion was completed in most cases between 4 and 12 hours after the bite, which may negatively influence prognosis by facilitating symptom progression. Previous studies have demonstrated that treatment delay is strongly associated with increased envenomation severity^14^ ^,^ ^24^ ^,^ ^28^ ^,^ ^38^ ^,^ ^39^.

Renal involvement was documented in both *Bothrops* and *Crotalus* envenomations, consistent with previous reports describing the nephrotoxic potential of these venoms; nephrotoxicity occurs through multifactorial mechanisms, including direct cytotoxicity, hemodynamic alterations, and myoglobinuria^40^ ^,^ ^41^. In the present study, renal complications occurred more frequently among patients who had longer intervals between the bite and antivenom infusion (>75th percentile). Similar findings have been reported elsewhere, identifying acute kidney injury as a major determinant of morbidity and mortality in snakebite victims, particularly those with delayed administration of antivenom therapy^6^ ^,^ ^11^ ^,^ ^42^. Early administration of antivenom reduces the incidence and severity of acute kidney injury, thereby improving patient prognosis^10^ ^,^ ^32^ ^,^ ^43^. The criticality of timely administration of antivenom for preventing severe renal complications, including the need for renal replacement therapy, has been emphasized in comparable studies^10^ ^,^ ^44^.

The overall mortality was 1.2%, with an even distribution between *Bothrops* and *Crotalus* envenomations. The crude analysis showed that longer intervals between the bite and antivenom administration were associated with an increased risk of death; however, this association lost significance after the adjusted analysis. These findings are consistent with those of previous studies, in which delays of >6 hours in administering antivenom therapy were reported to be associated with greater envenomation severity and higher mortality^29^. Other studiesindicate that a high number of snakebite deaths occur in regions with limited access to healthcare resources, such as remote or rural areas, largely due to delayed antivenom administration^18^ ^,^ ^45^ ^,^ ^46^.

Beyond the clinical implications of envenomation, the interval between the bite and antivenom administration may also be a marker of social inequalities and healthcare access. Populations living in rural areas or regions with limited transportation and healthcare infrastructure are more likely to experience delays, which can significantly impact clinical outcomes^39^ ^,^ ^47^. The presence of structural barriers highlights that the time to completion of antivenom infusion should be interpreted as both a factor reflecting clinical severity and an indicator of social vulnerability and disparities in healthcare access. Addressing these challenges requires integrated toxicovigilance strategies, strengthening of regional antivenom distribution, and improvements in transportation and healthcare logistics in high-risk areas.

These inequalities, particularly evident in developing countries, emphasize that delays in care extend beyond clinical factors and reflect structural disparities in access to healthcare. The findings of this study are consistent with those reported in African and Asian countries, where limited access to healthcare facilities and delayed antivenom administration were major determinants of morbidity and mortality^47^ ^,^ ^48^. Experiences from Nigeria, India, and Sri Lanka highlight similar challenges, including long distances from remote areas to referral centers, unequal antivenom distribution, and variability in clinical management protocols^47^ ^,^ ^49^ ^,^ ^50^. These studies emphasize the global significance of snakebite envenoming as a neglected tropical disease and reinforce the need to strengthen epidemiological surveillance and establish effective antivenom distribution and clinical care pathways. Within the Brazilian context, although structural inequalities persist, the operation of centers such as CIATox represent important advances in reducing mortality and improving clinical management.

Although the association between the time to antivenom administration and clinical outcomes is well established, multiple barriers continue to impede timely access to treatment in Brazil. These include the still centralized distribution of antivenoms, logistic limitations in maintaining the cold chain, and transportation challenges that delay delivery to remote areas. Insufficient technical training and limited coverage of snakebite management in health professional education also contribute to delays in case recognition and appropriate treatment^39^ ^,^ ^45^ ^,^ ^46^ ^,^ ^51^ ^,^ ^52^.

In conclusion, the retrospective study involving a cohort assembled over 8 years provides relevant epidemiological and clinical insights into snakebite envenomations in northern Paraná. Longer intervals between the bite and specific antivenom administration were significantly associated with increased risks of renal complications and death, and the association with mortality persisted after adjusting for epidemiological and clinical variables. These findings highlight that time to antivenom infusion remains a critical and modifiable determinant of outcomes. Despite generally appropriate clinical management, delays in antivenom administration continue to challenge effective care. Strengthening strategies that expand access, decentralize antivenom distribution, and enhance the training of healthcare professionals is essential to reduce preventable complications and fatalities. The standardized care at CIATox-Londrina ensures data consistency and enables risk factors for adverse outcomes to be identified. Limitations included the retrospective design, potential underreporting or incomplete data, and small number of rare events, which may have affected the statistical power of the study, particularly for outcomes such as acute kidney injury. Although the findings reflect the CIATox-Londrina population, regional differences may limit generalization. Nevertheless, the results provide valuable evidence that supports optimizing clinical protocols, preventive measures, and healthcare planning for snakebite management.

## Data Availability

Data will be made available on request.
